# Determination of the Cadmium, Chromium, Nickel, and Lead Ions Relays in Selected Polish Medicinal Plants and Their Infusion

**DOI:** 10.1007/s12011-017-1072-5

**Published:** 2017-06-15

**Authors:** Janusz Mirosławski, Andrzej Paukszto

**Affiliations:** Department of Environmental Engineering and Occupational Hygiene, Occupational Safety Management in Katowice, Bankowa 8, 40-007 Katowice, Poland

**Keywords:** Heavy metals, Herbs, Infusion, AAS, Safe therapy

## Abstract

Concentration of Cd, Cr, Ni, and Pb were determined in peppermint leaves (Folium Menthae piperitae), chamomile blossom (Anthodium Chamomillae), and their infusions by atomic absorption spectrometry (AAS) after microwave digestion of plants samples. Peppermint and chamomile preparations by five different producers were purchased at various pharmacies in Poland. Infusions were made from herbs according to prescription for patients, provided by the producer of medicine on the package. Results show that in exam of medicinal plants the content of cadmium exceeds World Health Organization (WHO) standards. The highest level of extraction was observed for Ni (50–70% of the total content in herb), Cr (28–36%), Cd (14–16%), and the lowest for Pb (about 6%). The calculated daily intake all analyzed elements was very low. Phytotherapy with using of these herbs is safe for health of patient even in case of long time therapy.

## Introduction

Traditional medicine (TM) is an important and often underestimated part of health services. In some countries, traditional medicine may be termed as complementary medicine (CM). A WHO support TM [1]. In recent years, we have witnessed the increasing growth in popularity of medicinal products from plants in many countries [2]. Plants which are won for medical purposes are subject to anthropopression. The content of various chemical compounds in plants is not constant and it changes depending on climate, soil types, harvest season, and environmental pollution. Medicinal plants are able to accumulation of metals, both essential to life, and toxic for man [3]. Persons who use during long-time herbal preparations with to big content of microelements in raw plants can have unwanted effects [4, 5]. In the scientific literacy, it exists a lot of reports about content of heavy metals in medicinal herbs [6–12], whereas there are few reports about content of these metals in prepared infusion of medicinal herbs [13–18]. The most common heavy metal pollution listed by the Environment Protection Agency (EPA) are arsenic, cadmium, chromium, copper, mercury, nickel, lead, and zinc [19]. Chromium and nickel play a physiological role in the human body and are present therein in trace amounts. Chromium content is approximately 6 mg/70 kg of body weight, and its daily intake is 0.06 up to 0.29 mg. These values for nickel are, respectively, 10 mg/70 kg of body weight and 0.45 mg [20]. Cadmium and lead show no physiological role in the human body. Cadmium content is approximately 0.05 mg/70 kg of body weight and its daily intake is 0.01 to 0.02 mg. For lead, these values are, respectively, 0.12 mg/70 kg of body weight and 0.1 up to 0.5 mg [20].

The development of various branches of industry and increased demand for metals have caused not only greater exposure of people working in industry, but it has also increased exposure of the general population to, among others, chromium, cadmium, nickel, and lead. Distant effects caused by chromium compounds are carcinogenic, mutagenic, embryotoxic, and teratogenic effects. The processes of reducing hexavalent chromium to trivalent chromium that take place inside cells can be considered an activation of carcinogenic properties of chromium as they increase the probability of trivalent chromium interaction with DNA [20]. Cadmium can cause changes in genetic material, especially in mammalian cell chromosomes. Kidneys are critical organs in which cadmium is well cumulated. The International Agency for Research on Cancer classifies nickel compounds in Group 1—a carcinogenic substance. The toxic effect of lead on the human body is shown in hematopoietic disorders, hemic acid synthesis, inhibition of hemoglobin synthesis, and shortening of red blood cell life. At later stages, reticulocytosis and anemia are reported. Moreover, lead damages the nervous system, renal functions, and causes digestive system disorders [20]. The herbal material used by producers comes from both industrial crops and from individual suppliers whose herbal material can be contaminated with heavy metals.

The aim of this work was to determine the content of cadmium, chromium, nickel and lead in Anthodium Chamomillae (blossom), Menthae piperitae (folium), and in the infusions prepared from these plants as well to determine the risk of poisoning through these metals the patients during a long-time therapy. The chamomile flower and the peppermint leaf are the most commonly used herbal preparations in Poland due to the wide range of their therapeutic effects, which was the reason for choosing these herbs for research

## Experimental

### Materials

The sample materials were five preparations of Anthodium Chamomillae and five preparations of peppermint prepared by various Polish producers. The studied preparations of the chamomile flower and the peppermint leaf were produced by the following Polish producers:

- DR VITA Sp. z o.o.
- Krakowskie Zakłady Zielarskie
- SYNOPTIS PHARMA Sp. z o.o.
- Poznańskie Zakłady Zielarskie
- HERBAPOL Lublin S.A.

All preparations were in the form of brewing bags.

All preparations were obtained from five different pharmacies, which guaranteed different delivery batches and the preparations had valid usability dates. Individual samples of each manufacturer were represented by three samples taken from three different packages.

### Reagents

It was used in researches following reference material: mixture of Polish herbs (INC-MPH-2) produced by Analytical Chemistry Unit at the Institute of Chemistry and Nuclear Technology. It was used spectrally pure standards of mass concentration for Cd, Cr, Ni, and Pb with concentration 1 mg ml^−1^ produced by Physical Chemistry Department in Central Office of Measures, as well concentrated (65%) spectrally pure nitric acid (V) produced by J.T. Baker and purified water.

### Apparatus

It were used following devices: microwave oven Magnum II produced by ERTEC-Poland, analytical balance WAX 110 produced by Precision Engineering Facility RADWAG Poland, spectrometer for atomic absorption spectroscopy AAS-3 produced by Carl-Zeiss Jena, Germany.

### Procedure

The samples of herbs in amount of 0.3 g were submitted to mineralization process in the microwave oven using concentrated nitric acid (V) [13]. Program of the process is presented in the Table 1.

**Table 1:** Parameters for the microwave digestion

Step	Time (min.)	Temperature (°C)		Pressure (bar)	
		Min.	Max.	Min.	Max.
1	2.30	280	285	17	20
2	6.30	285	290	42	45

Received digest solutions were transferred quantitatively to graduated flasks and completed with purified water to volume of 25 ml. Infusions of researched herbs were prepared according to recipe which is placed by the producer on the package through flooding 200 ml of purified, boiled water determined mass of herbal preparation (ca. 2 g). Ten minutes after cooling, the infusion was strained under vacuum from solid phase by using of funnel with fritted glass G3. Separated phase was drier in temperature 20 °C to solid mass and submitted mineralization according to described procedure. In received digest solution were determined concentrations of Cd, Cr, Ni, and Pb using AAS method.

### Determining of Concentration of Cd, Cr, Ni, and Pb

The concentration of metal ions was determined with flame atomic absorption with deuterium correction of background in flame acetylene–air. Measurement settings are presented in the Table 2.

**Table 2:** Parameters for element determination by the AAS method

Element	Wavelength (nm)	Slit wild (mm)	Lamp current (mA)
Cd	228,8	0,3	4
Cr	357,3	0,2	4
Ni	232,0	0,25	4
Pb	217,0	0,4	5

The accuracy of analytic procedure was checked out through the analysis of certified reference material Mixed Polish Herbs (INCT-MPH-2). Received results are presented in the Table 3.

**Table 3:** Certified and determined value

Element	Certified value ±SD (mg kg^−1^)	Determined value ±SD (mg kg^−1^)	% recovery
Cd	0.199 ± 0.015	0.186 ± 0.015	93.5
Cr	1.69 ± 0.13	1.78 ± 0.14	105.3
Ni	1.57 ± 0.16	1.47 ± 0.14	93.6
Pb	2.16 ± 0.23	2.14 ± 0.25	99.1

### Determining the Daily Dosage of Cd, Cr, Ni, and Pb

Daily dosage of metals taken by the patient during the therapy with the analyzed herbs was determined basing on the following formula:

$$ D=\left({c}_1\hbox{--} {c}_2\right)\cdotp m $$

*C*_1_: content of metal in ther herb (mg kg^−1^), (Table 4)
*C*_2_: content of metal in the herb after infusion (mg kg^−1^), (Table 5)
*m*: amount of herb in one sachet (kg), (on average 0.002 kg)

**Table 4:** The concentrations of Cd, Cr, Ni, and Pb in raw material: chamomile blossom and peppermint leaves (mg kg^−1^) ± SD (*n* = 3)

Number of producer	Chamomile blossom				Peppermint leaves			
	Cd	Cr	Ni	Pb	Cd	Cr	Ni	Pb
1	0.22 ± 0.02	1.14 ± 0.15	2.24 ± 0.31	0.48 ± 0.10	0.47 ± 0.06	1.26 ± 0.18	2.36 ± 0.42	0.92 ± 0.12
2	0.19 ± 0,02	1.81 ± 0.21	2.81 ± 0.42	0.65 ± 0.11	0.46 ± 0.05	2.15 ± 0.21	1.15 ± 0.26	0.78 ± 0.11
3	0.18 ± 0.02	1.14 ± 0.16	2.14 ± 0.34	0.44 ± 0.08	0.51 ± 0.04	0.95 ± 0.14	2.20 ± 0.23	0.46 ± 0.07
4	0.20 ± 0.02	1.05 ± 0.12	2.05 ± 0.28	0.70 ± 0.12	0.72 ± 0.07	0.92 ± 0.22	2.75 ± 0.31	0.82 ± 0.11
5	0.32 ± 0.03	1.22 ± 0.15	2.22 ± 0.30	0.85 ± 0.21	0.73 ± 0.07	1.75 ± 0.28	3.48 ± 0.47	1.11 ± 0.13
Average	0.22 ± 0.06	1.27 ± 0.31	2.29 ± 0.30	0.62 ± 0.18	0.58 ± 0.14	1.41 ± 0.53	2.38 ± 0.85	0.82 ± 0.24

**Table 5:** The concentrations of Cd, Cr, Ni and Pb in: chamomile blossom and peppermint leaves remaining after infusion (mg kg^−1^) ± SD (*n* = 3)

Number of producer	Chamomile blossom				Peppermint leaves			
	Cd	Cr	Ni	Pb	Cd	Cr	Ni	Pb
1	0.19 ± 0.02	1.14 ± 0.16	0.99 ± 0.14	0.44 ± 0.08	0.41 ± 0.08	0.82 ± 0.11	0.71 ± 0.21	0.87 ± 0.12
2	0.15 ± 0.03	1.32 ± 0.21	1.12 ± 0.14	0.61 ± 0.09	0.39 ± 0.06	1.29 ± 0.15	0.25 ± 0.18	0.75 ± 0.12
3	0.15 ± 0.02	0.82 ± 0.18	0.88 ± 0.11	0.42 ± 0.07	0.44 ± 0.06	0.64 ± 0.12	0.73 ± 0.15	0.44 ± 0.10
4	0.17 ± 0.02	0.75 ± 0.15	0.82 ± 0.11	0.65 ± 0.07	0.62 ± 0.08	0.60 ± 0.14	0.85 ± 0.16	0.75 ± 0.12
5	0.27 ± 0.04	0.89 ± 0.13	0.87 ± 0.15	0.81 ± 0.10	0.65 ± 0.10	1.10 ± 0.21	1.15 ± 0.22	1.04 ± 0.25
Average	0.19 ± 0.05	0.98 ± 0.24	0.94 ± 0.12	0.59 ± 0.16	0.50 ± 0.12	0.89 ± 0.30	0.74 ± 0.32	0.77 ± 0.22

Assuming, in accordance with the producer’s recommendations that the patient takes once daily the infusion from one sachet, the obtained result is the daily dosage of a taken metal.

## Results and Discussion

The concentration of Cd, Cr, Ni, and Pb in investigated herbal plants is presented in Table 4. The concentration of these metals in their infusion was LOD (LOD for Cd is 0.05 mg kg^−1^, for Cr and Ni is 0.15 mg kg^−1^, for Pb is 0.20 mg kg^−1^). The results obtained show that examined medicinal herbs contained in the mg kg^−1^ range varied widely.

The concentrations of Cd, Cr, Ni, and Pb in herbal plants remaining after infusion are given in Table 5.

Researches proved that the contents of determined elements (mg kg^−1^) are varied in preparations of various producers:

- Chamomile blossom—Cd: 0.18–0.3; Cr: 1.05–1.81; Ni: 2.05–2.81; Pb: 0.44–0.85.
- Peppermint leaves—Cd: 0.46–0.73; Cr: 0.92–2.15; Ni: 1.15–3.48; Pb: 0.46–1.11.

Similar results of Cd, Cr, Ni, and Pb contents in chamomile preparations were obtained by other authors and were, respectively, in mg kg^−1^: 0.44, 1.22, 1.80, and 0.72. Those authors also obtained a very similar degree of nickel infusion for this preparation equal to 54.5%. The studied preparations were from Turkey [21]. In turn, researchers from Lebanon identified the content of Cr, Cd, and Pb in 16 different herbal preparations found in Lebanon, resulting in the following average values of mg kg^−1^, respectively, 2.96, 17.4, and 16.5 [22]. Researchers from Saudi Arabia determined the following contents in mg kg^−1^ for Cd, Cr, Ni, and Pb in mint formulations were 0.075, 2.44, 1.33, and 0.825; and for chamomile preparations, respectively, 0.12, 1.32, 1.24, and 1.03 [23]. These results are similar to those obtained in our research.

The presented results are similar to the ones obtained by the researchers in other countries (e.g., Saudi Arabia, Lebanon, Turkey), who determined similar values for various herbs, also chamomile and peppermint leaves. It must be stressed that the level of content of a given element in a plant is the consequence of many parallel, territorially differentiated factors: type of soil, amount of precipitation, size of athropopressure, pH environment, chemical forms of the occurrence of these elements both in soil and in plant, and origin of the herb (natural collection or from farmlands).

Higher content of Cd, Cr, Ni, and Pb were found in peppermint leaves in comparison with preparations of chamomile blossom; on the average: for Cd by 163%, Cr—11%, Ni—4%, and Pb—32%.

There are, however, no standards for medicinal plants raw plant materials witch establish a permissible levels in raw plant material only for arsenic, cadmium, and lead, amounting to 1.0, 0.3, and 10.0 (μg kg^−1^), respectively (WHO 1998).

Our studies have demonstrated that Polish medicinal plants the content of cadmium exceeds (peppermint leaves—all of producers and chamomile blossom—one producer) WHO standards. The content of Pb in the herbs analyzed is lower than permissible WHO level—10 μg kg^−1^ dry weight. On the grounds of comparison of metals content in herbal material before and after preparing of infusions the efficiency of extraction (% of total metal contents) for each elements was different and similar for both researched herbs of all five producers—Table 6. As seen from the Table 6, the highest efficiency of extraction is found for Ni—on average for chamomile blossom 59.1%, for peppermint leaves—70.1%, and the lowest for Pb—, respectively 6.2 and 5.7%. It results most likely from differences in solubility in water of Ni (II) compounds in comparison with Pb (II). On the grounds of metals content in raw material and after infusion, considering recipe for preparing of infusions, 200 ml infusion daily, mass of herb which is included in one received dose (on average ca. 2 g); it was calculated that the quantity of heavy metals which is delivered to the organism in this way during phytotherapy is significantly lower than maximal doses, established by European Food Safety Authority (EFSA). Calculated doses of defined metals for researched preparations which are received once daily with infusion are presented in the Table 7

**Table 6:** Infusion (% of total mount) ± SD (*n* = 3)

Number of producer	Chamomile blossom				Peppermint leaves			
	Cd	Cr	Ni	Pb	Cd	Cr	Ni	Pb
1	13.6 ± 0.98	29.8 ± 1.15	55.8 ± 1.30	8.3 ± 0.21	12.8 ± 0.60	34.9 ± 1.07	69.1 ± 1.05	5.4 ± 0.26
2	21.1 ± 0.55	27.1 ± 1.05	60.1 ± 2.42	6.2 ± 0.16	15.2 ± 0.62	40.0 ± 1.10	78.3 ± 1.15	3.8 ± 0.20
3	16.7 ± 0.78	28.1 ± 1.20	58.9 ± 1.87	4.5 ± 0.17	13.7 ± 0.55	32.6 ± 1.01	66.8 ± 1.11	4.3 ± 0.31
4	15.0 ± 0.95	28.6 ± 1.22	60.0 ± 2.05	7.1 ± 0.19	13.9 ± 0.58	34.8 ± 1.12	69.1 ± 1.13	8.5 ± 0.42
5	15.6 ± 0.78	27.0 ± 0.98	60.8 ± 2.15	4.7 ± 0.16	11.0 ± 0.51	37.1 ± 1.14	67.0 ± 1.22	6.3 ± 0.38
Average	16.0 ± 2.85	28.1 ± 1.16	58.1 ± 1.98	6.2 ± 1.61	13.9 ± 1.56	35.9 ± 2.80	70.1 ± 4.74	5.7 ± 1.86

**Table 7:** Daily doses of Cd, Cr, Ni, and Pb (μg/daily)

Medicine plant	Cd	Cr	Ni	Pb
Chamomile blossom	0.07	0.07	1.52	0.08
Peppermint leaves	0.16	1.01	1.93	0.08

The Joint FAO/WHO Expert Committee on Food Additives established a provisional tolerable monthly intake of 25 μgCd/kg body weight, whereas the EFSA Panel on Contaminants in the Food Chain nominated a tolerable weekly intake of 2.5 μgCd/kg body weight to ensure sufficient protection of all consumers [24]. Mean lifetime dietary exposure was estimated at 0.68 μgPb/kg body weight per day in the European population based on middle bound mean lead occurrence. Exposure was highest for toddlers and other children with 1.32 and 1.03 μgPb/kg body weight per day, respectively, while the two infant surveys ranged between 0.83 and 0.91 μgPb/kg body weight per day. Adult exposure was estimated at 0.50 μgPb/kg body weight per day. The elderly and very elderly population groups had similar profiles to the adult age group, while adolescents had slightly higher estimated dietary exposure [25].

## Conclusions

Content of Cd, Cr, Ni, and Pb in preparations of chamomile blossom and peppermint leaves of various producers is varied and do not exceed values which are established by WHO. The highest efficiency of extraction was stated for Ni and Cr, the lowest for Pb. Doses of Cd, Cr, Ni, and Pb inserted into organism during phytotherapy with preparations of chamomile blossom and peppermint leaves are significantly below acceptable daily intake and are not danger for patients, even in case of long-term therapy with these preparations.

This research did not receive any specific grant from funding agencies in the public, commercial, or not-for-profit sectors.

The authors wish to thank Elżbieta Krawczyk-Neifar for her valuable help in preparing the English version of the text.
